# Performance of Laboratory Professionals Working on Malaria Microscopy in Tigray, North Ethiopia

**DOI:** 10.1155/2017/9064917

**Published:** 2017-12-19

**Authors:** Megbaru Alemu, Desalegn Tadesse, Tesfaye Hailu, Wondemagegn Mulu, Awoke Derbie, Tadesse Hailu, Bayeh Abera

**Affiliations:** ^1^Department of Microbiology, Immunology and Parasitology, Bahir Dar University, Bahir Dar, Ethiopia; ^2^Departments of Medical Parasitology and Vector Biology, Mekelle University, Mek'ele, Ethiopia; ^3^School of Public Health, Mekelle University, Mek'ele, Ethiopia

## Abstract

**Background:**

Microscopic analysis of stained blood smear is the most suitable method of malaria diagnosis. However, gaps were observed among clinical laboratory professionals in microscopic diagnosis of malaria.

**Methods:**

A cross-sectional study was conducted in December 2015 among 46 laboratory professionals. Data was collected via on-site assessment and panel testing. The slide panel testing was composed of positive and negative slides. The kappa score was used to estimate the agreement between participants and reference reader.

**Results:**

The overall agreement between the study participants and the reference reader in malaria detection was 79% (kappa = 0.62). Participating in refresher training on malaria microscopy (Adjusted Odds Ratio (AOR = 7, CI = 1.5–36.3)) and malaria epidemic investigation (AOR = 4.1 CI = 1.1–14.5) had statistical significant association with detection rate of malaria parasites.

**Conclusion:**

Laboratory professionals showed low performance in malaria microscopy. Most of the study participants were graded “in-training” in laboratory diagnosis of malaria.

## 1. Introduction

Malaria is the major cause of morbidity and mortality in the developing world in which 80% cases and 90% deaths reside in the African continent [1]. In Ethiopia, approximately 68% of the total population lives in areas at risk of malaria. It is ranked as the leading communicable disease in the country, accounting for about 30% of the overall disability adjusted life years lost. In the past years, it has been consistently reported as one of the three leading causes of morbidity and mortality [2, 3]. As in other areas of Ethiopia, malaria is unstable in Tigray, making the region prone to epidemics with high morbidity and mortality in all age groups. Furthermore, as malaria transmission strikes during the planting and harvesting season, it adversely affects food security and impoverishes and isolates affected communities [4].

One of the main approaches of malaria control relays on early diagnosis followed by timely treatment. Owing to lack of specificity of signs and symptoms of malaria, the application of clinical diagnosis is only limited to areas lacking laboratory facilities for malaria diagnosis [4, 5]. Giemsa stained blood smear microscopy is the mainstay of malaria diagnosis [6]. Quantitative results for the evaluation of the extent of parasitemia clearance can be attained by this diagnostic approach. It is a simple, quick, low-cost technique that enables the correct diagnosis of malaria parasites and the determination of parasite density. However, the lack of qualified professionals to correctly diagnose malaria and the lack of quality control in the laboratory diagnostic process have challenged the current strategy to control malaria elsewhere in the world [4]. Quality control programs are a prerequisite of competent microscopy. The World Health Organization (WHO) recommends the cross-checking of blood slides: in such programs, a sample of blood slide is sent to the reference laboratory and it is checked for accuracy, and then feedback is sent to the peripheral laboratories [2].

However, little efforts were made so far to precisely determine and identify sources of error in microscopic diagnosis and quantification of parasitemia and to secure quality control program for malaria microscopy [7]. Consequently data on the current situations of laboratory logistics, procedures, and proficiency of laboratory professionals with respect to microscopic investigation of malaria parasites is lacking in Ethiopia.

Therefore, the current study was aimed at assessing the general laboratory logistics and performance of laboratory professionals on malaria microscopy in Tigray, North Ethiopia.

## 2. Methods

### 2.1. Study Design

Institution based cross-sectional study was conducted among selected laboratory service providers of Tigray from December 2015 to August 2016. The study area is located 586 kilometers far from the capital Addis Ababa. The mean annual temperature ranges from 16°C to 22°C and the altitude ranges from 1200 to 2400 meters above the sea level. The annual rain fall is 668 mm. During the time of data collection, there were a total of 61 laboratory professionals working in thirty-two governmental health centers and three hospitals. Most of the laboratories provide microscopic examination of urine sediment, blood film, stool, acid fast bacilli staining, and gram staining routinely. Malaria diagnosis is mainly based on blood film examination and Rapid Diagnostic Tests (RDTs) in the health posts. Laboratory professionals working at the selected health institutions on malaria microscopic diagnosis were the study population.

### 2.2. Sample Size & Sampling

The sample size was determined using single population proportion estimate (*z*2*p*(1 − *p*)/*d*2) considering 95% confidence level, 0.5 proportion, assuming that malaria microscopy in 50% of the health care laboratories is below the standard and marginal error of (5%). Thus, a sample size of 384 was calculated. However, the expected total sample size in the selected target areas is less than the calculated sample size (*N* = 35); we adjusted our sample size based on the correction formula; *n* = *n*_0_/1 + (*n*_0_ − 1)/*N*. Therefore, a total sample size of 22 was calculated and all permanent/stable laboratory professionals engaged in malaria microscopy in the selected health institutions were involved.

### 2.3. Variables

Proficiency of laboratory professionals on malaria microscopy was the dependent variables while age, sex, service year, qualification, taking refreshment training, and availability of guidelines were the independent variables.

### 2.4. Data Collection

#### 2.4.1. On-Site Assessment

Data on demographic characteristics of participants and general set-up of the selected laboratories were collected via self-administered interview and on-site observation using structured checklist.

### 2.5. Malaria Slide Panel Testing

For the purpose of slide panel preparation, about 5 ml of venous blood was collected from confirmed malaria outpatients at Maytsebri Health Center. Negative slides on the other hand were prepared from apparently healthy persons who were also negative for malaria. The panel slides were prepared and validated by malaria microscopists at Tigray regional laboratory. Both thick and thin blood films were prepared. In the thick film preparation, three drops of blood were distributed over an area of 1 cm^2^. Thin smear on the other hand was prepared by evenly distributing a drop of blood on a grease free microscopic slide. Slides were labeled, dried, fixed with methanol alcohol (thin smear only), and stained (using 3% Giemsa stain solution for 30 minutes). The stained blood film was washed with distilled water and air dried. Detection and species identification of the plasmodium parasites were made via thick and thin blood films, respectively.

The panel testing comprised six samples. Slide panels A, B, C, and D contained* P. falciparum* ++++,* P. vivax *+,* P. falciparum* +, and* P. vivax* +++ in thin thick blood films, respectively. Slide panel E possessed both* P. falciparum* and* P. vivax*. Slide panel G possessed malaria negative blood films. A total of 276 blood films (6 for each participant) were distributed. Study participants were then requested to report the detection of malaria (negative, positive), Plasmodium species identification (*P. falciparum*,* P. vivax,* or Mixed), and grade parasitemia (semiquantitatively).

### 2.6. Statistical Analysis

Data were entered and analyzed using Statistical Package for Social Sciences (SPSS) version 20. Descriptive statistics were used to get summary measures. Performance of laboratory professionals on malaria microscopy was tested by computing the specificity, sensitivity, and negative and positive predictive values of their reports. Percent of agreement was determined by* kappa* score. Chi-square and logistic regression tests were employed to compare the performance of professionals with demographic variables and other factors. *P value* of <0.05 was considered statistical significant.

### 2.7. Operational Definition

#### 2.7.1. Expert

It includes participants who scored ≥90% agreement with reference reader in the detection, speciation, and quantification of malaria parasites.

#### 2.7.2. Reference

It icludes participants who scored ≥80% but <90% agreement with reference reader in the detection, speciation, and quantification of malaria parasites.

#### 2.7.3. Competent

It includes participants who scored ≥70% but <80% agreement with reference reader in the detection, speciation, and quantification of malaria parasites.

#### 2.7.4. In-Training

It includes participants who scored <70% agreement with reference reader in the detection, speciation, and quantification of malaria parasites based on the recommendation of the World Health Organization [8].

#### 2.7.5. Major Error

Major errors include (i) incorrect diagnosis of malaria, that is, reporting false positive or false negative results, (ii) not mentioning the presence of* P. falciparum* (either reporting non-*falciparum *species in the case of* Plasmodium falciparum* or no species identification at all).

#### 2.7.6. Minor Error

Minor errors include (i) identification error of* P. vivax* and of a mixed infection (reporting single infection in case of mixed parasites) and (ii) reporting a parasite density differing one “+” from the reference result [9].

## 3. Ethical Clearance

Ethical clearance was sought from the Ethics Review Committee of the College of Health Sciences at Mekelle University with the reference number (ERC055/2015). Informed written consent was secured from the participants and blood donors for slide panel preparation. Codes were provided for participants to maintain confidentiality of their report on malaria slides.

## 4. Results

### 4.1. Demographic Characteristics of Study Participants

A total of 46 clinical laboratory professionals were enrolled in the study. The mean age of the participants was 28.9 years. Males accounted for 32 (69.6%) of the participants. Majority of the participants served for less than five years in the diagnostic centers (50%). About 39% of the participants took refreshment trainings on malaria microscopy whereas more than half (58.7%) of the participants had participated in malaria epidemic investigations (Table 1).

### 4.2. On-Site Assessment

Out of the total 22 laboratories inspected, 7 (30.4%), 10 (43.5%), and 5 (22.7%) had one, two, and three functional microscopes, respectively. Majority (52.2%) of laboratories employed Binocular with external light type of microscope.

Most (87%) of the laboratories had guideline for preparation of working reagents. Giemsa stain was the only kind of stain being utilized for malaria microscopy. Similarly, all the clinical laboratories were utilizing new microscopic slides for individual patients (i.e., no reuse of slides). Most of the inspected laboratories (91.3%) were using semiquantitative (plus system) of quantification to count parasites (Table 2). Drying racks accompanied all malaria laboratories.

### 4.3. Panel Testing

The overall sensitivity, specificity, PPV and NPV of laboratory professionals in detection, and species identification of malaria parasites were 63% and 95.7%, 93.6% and 72.1%, and 78.3%, 95.7%, 94.4%, and 81.5%, respectively (Table 3). Out of the total 46 laboratory personnel involved in the study, only 1 (2.2%), 2 (4.3%), and 7 (15.2%) of the participants correctly reported all the six, five, and four slides, respectively (Table 3).

Sixty-three percent of the participants correctly reported the species of slide A but only 10.9% of them correctly reported the parasite density (++++). Similarly 30.4% of the participants correctly reported the species of slide B; however, only 15.2% were able to report the parasites density. Slide C contains* Plasmodium falciparum* (+) and only 6.5% and 2.2% of the participants correctly reported the species and parasite density, respectively. Mixed infections of* Pf* and* Pv* (Slide E) were correctly reported only by 15.2% of the participants. About 10% of laboratory professionals have reported negative slides (slide G) as false positive* P. falciparum*. Nearly 35% participants committed major error for falsely reporting* P. vivax* species in the case of* P. falciparum *infection. Similarly, about half of the participants in our study committed minor error in reporting mixed infections (Table 3).

As depicted in Table 3, the overall agreement between the study participants and the reference reader in the detection, speciation, and quantification of malaria parasites was 79% (*k* = 0.62), 87% (*k* = 0.58), and 65% (*k* = 0.55), respectively. The lowest sensitivity (21.7%) and agreement (59%) were observed on mixed infections. The detection rate of laboratory professionals was better for* P. vivax *(63%) than* P. falciparum *(56.5%).

### 4.4. Multivariate Analysis on Proficiency of Malaria Microscopy

On regression analysis, taking refresher training on malaria microscopy and participation in malaria epidemic investigation had significant association with proficiency of laboratory personnel on malaria microscopy. Laboratory personnel who took refresher training were 7 times better in detection and identification of malaria parasites compared to those who did not take refresher training (AOR = 7, CI = 1.5–36.3). Similarly those laboratory personnel who participated in malaria epidemic investigation were 4.1 times better than those who did not participate in epidemic investigation in detection and identification of malaria parasites (AOR = 4.1, CI = 1.1–14.5) (Table 4).

## 5. Discussion

In Ethiopia diagnostic and treatment health facilities employed microscopic diagnosis of malaria using Giemsa stain. However, there is disparity in detection rate and speciation of malaria parasites among different health institutions which might be related with the qualification and experience of medical laboratory professionals. Therefore, this study showed the first performance evaluation of laboratory professionals on malaria microscopy in diagnostic centers of Tigray and needs to be considered by Federal Ministry of Health for future national study and action.

This study showed the presence of adequate staining reagents, microscopic slides, drying racks, and guidelines for reagent preparation. However, only 58% and 39.1% of the participants participated in epidemic investigation and refresher trainings. This finding is in sharp contrast with previous study done in Northwest Ethiopia [10].

The overall sensitivity and specificity of laboratory professional reports in detecting malaria parasites were 63% and 95.7%, respectively. The sensitivity was lower than findings from Hawassa (82%) [11], Addis Ababa (65.7%) [12], and Zambia (88%) [13]. The lower sensitivity in parasite detection in this study implies high proportion of false negative results and hence underreport of true infections. The specificity (95.7%) in the present study was in agreement with reports from Hawassa (96.5%), Ethiopia [11]. However, it was higher than reports elsewhere [12, 13].

In this study, the proportion of false negativity was higher in* P. falciparum* positive slides (43.5%) than* P. vivax* positive slides (37%). This contradicted a study from Hawassa, Ethiopia, where false negativity was higher in* P. vivax *[12]. Failure rate in identification of* P. falciparum *in this study (43.5%) was higher than a report from Ethiopia (40.4%) [11], Canada (27%) [14], and USA (39%) [15]. This higher failure rate of* P. falciparum* identification could be due to the predominance of* P. vivax *in the area. The overall agreement between participants and reference readers in detection (*K* = 0.62), species identification (*K* = 0.58), and quantification of the plasmodium (*K* = 0.55) in the present study was inconsistent with previous reports from south Ethiopia [11], Addis Ababa [12], and Hong Kong [16].

Nearly half of the participants in the present study reported mixed infections wrongly as* P. vivax* monoinfection. It was in sharp contrast with previous studies [11, 12] in which participants reported mixed infections as* P. falciparum* monoinfection. This higher rate of failure in reporting mixed infections might be due to the fact that most study participants were not taking sufficient refreshment training on malaria microscopy, similar to that reported somewhere else in Ethiopia [11, 12]. The hypoendemic transmission of malaria in the area might also have contributed to the lower performance of lab professionals in malaria diagnosis [4].

In this study lower agreement on parasite detection and identification of slides with low parasitic density and mixed infection were observed. Moreover, the performance of laboratory professionals in* P. falciparum* identification was better at high density parasitemia (66.7%) slides than the lower density (50%). This was coherent with previous studies in other parts of Ethiopia [11, 12]. However, the overall agreement in detection of* P. falciparum* parasites on high density parasitemia was lower than previous reports elsewhere [11, 12, 14, 16].

Participating in malaria epidemic investigation and refresher training on malaria microscopy were independently associated with a better performance of malaria microscopic diagnosis in our study. It is in line with study from Congo [9].

The current study addressed the performance of clinical laboratory professionals on malaria microscopy using stained malaria slides. Hence the extent to which professionals stain malaria blood films was not assessed and it was taken as the main limitation of this study.

## 6. Conclusions

Despite availability of consumables for malaria microscopy, professionals showed low performance in malaria microscopy. Based on WHO recommendation most of them in this study were graded “in-training” both in detection and in species identification of malaria parasites. Participants were better in species identification than in parasite detection. They showed the poorest agreement in the detection of mixed infections and low density* P. falciparum *parasitemia. Hence refreshing trainings should be delivered for the professionals periodically on malaria microscopy.

## Figures and Tables

**Table 1:** Demographic characteristics of the study participants' in Tigray, North Ethiopia, 2016.

Characteristics	Categories	Frequency	Percent
Gender	Male	32	69.6
	Female	14	30.4

Age (Yrs)	20–30	31	67.4
	31–40	12	26.1
	41–50	3	6.5

Educational status	College diploma	20	43.5
	University degree	26	56.5

Work experience (Yrs)	≤5	23	50.0
	6–10	16	34.8
	11–15	5	10.9
	≥16	2	4.3

Participation in malaria epidemic investigation	Yes	27	58.7
	No	19	41.3

Participation in refresher training on malaria microscopy	Yes	18	39.1
	No	28	60.9

**Table 2:** General laboratory setups of selected health institutions in Tigray, North Ethiopia, 2016.

Variables		Frequency	Percent
Number of microscope (s) in each lab	One	7	30.4
	Two	10	43.5
	≥Three	5	22.7

Presence of guideline for reagent preparation	Yes	19	86.4
	No	3	13.6

Kind of blood film used	Thin only	0	0.0
	Thick only	3	13.6
	Both	19	86.4

Internal quality control programs conducted	Yes	19	86.4
	No	3	13.6

Type of microscope	Binocular with inbuilt lamp	10	45.5
	Binocular with external light	12	54.5

Method of parasite quantification	Plus system	20	91.0
	WBC system	2	9.0

Average time to read a slide (minutes)	0–20	20	91.0
	21–40	2	9.0

**Table 3:** Performance of laboratory personnel to correctly identify and quantify malaria slides, Tigray, North Ethiopia, 2016.

Participant reader		Expert reader		Sensitivity	Specificity	PPV	NPV	Agreement	Kappa
		Pos	Neg						
Species identification									
*P. falciparum* (*n* = 92)	Pos	52	2	56.5%	95.7%	98.7%	26.8%	76%	0.61
	Neg	40	44						
	Total	92	46						

*P. vivax* (*n* = 92)	Pos	58	2	63.0%	95.7%	98.9%	30. 1%	79%	0.62
	Neg	34	44						
	Total	92	46						

Mixed infection (*n* = 46)	Pos	10	2	21.7%	95.7%	96.8%	16.9%	59%	0.52
	Neg	36	44						
	Total	46	46						

Parasitemia grading									
*Pf* ++++ (high density) (*n* = 46)	Pos	4	2	_	_	_	_	52%	0.49
	Neg	42	44						
	Total	46	46						

*Pf* + (low density) (*n* = 46)	Pos	2	2	_	_	_	_	50%	0.48
	Neg	44	44						
	Total	46	46						

**Table 4:** Multivariate analysis of malaria diagnosis using light microscopy in Tigray, North Ethiopia, 2016.

Characteristics		*N* (%)	Distributed slides (*n* = 276)		*P* value	AOR (95% CI)
			Correct (%)	Incorrect (%)		
Gender	Male	32 (69.6)	144 (66.7)	72 (33.3)	0.33	2.0 (0.1–2.1)
	Female	14 (30.4)	30 (50.0)	30 (50.0)		
Age (in years)	20–30	31 (67.4)	42 (46.7)	48 (53.3)	0.11	0.4 (0.1–1.3)
	31–50	15 (32.6)	132 (71.0)	54 (29.0)		
Educational level	Diploma	20 (43.5)	42 (41.2)	60 (58.8)	0.25	2.0 (0.6–6.8)
	Degree	26 (56.5)	102 (58.6)	72 (41.4)		
Work experience (in years)	1–10	39 (84.8)	144 (60.0)	96 (40)	0.56	3.3 (0.4–31.3)
	11–25	7 (15.2)	30 (83.3)	6 (16.7)		
Participation in epidemic investigation	Yes	27 (58.7)	120 (76.9)	36 (23.1)	0.026	4.1 (1.1–14.5)
	No	19 (41.3)	54 (45.0)	66 (55.0)		
Participation in refresher training	Yes	18 (39.1)	84 (87.5)	12 (12.5)	0.012	7 (1.5–36.3)
	No	28 (60.9)	90 (50.0)	90 (50.0)		
Average time	1–20	40 (87)	108 (60.0)	72 (40.0)	0.071	1.5 (04–5.3)
	21–40	6 (13)	66 (68.6)	30 (31.3)		
